# Traditional Chinese medicine and its active substances reduce vascular injury in diabetes via regulating autophagic activity

**DOI:** 10.3389/fphar.2024.1355246

**Published:** 2024-03-04

**Authors:** Yankui Gao, Lei Zhang, Fei Zhang, Rong Liu, Lei Liu, Xiaoyan Li, Xiangdong Zhu, Yonglin Liang

**Affiliations:** ^1^ Department of Basic Medicine, Gansu University of Traditional Chinese Medicine, Lanzhou, China; ^2^ Department of Traditional Chinese Medicine, Fujian University of Traditional Chinese Medicine, Lanzhou, China; ^3^ Department of Traditional Chinese Medicine, Jiangxi University of Traditional Chinese Medicine, Nanchang, China; ^4^ Department of Traditional Chinese Medicine, Ningxia Medical University, Yinchuan, China

**Keywords:** diabetic vascular complications, autophagy, TCM formulations, herbal extracts, active compounds

## Abstract

Due to its high prevalence, poor prognosis, and heavy burden on healthcare costs, diabetic vascular complications have become a significant public health issue. Currently, the molecular and pathophysiological mechanisms underlying diabetes-induced vascular complications remain incompletely understood. Autophagy, a highly conserved process of lysosomal degradation, maintains intracellular homeostasis and energy balance via removing protein aggregates, damaged organelles, and exogenous pathogens. Increasing evidence suggests that dysregulated autophagy may contribute to vascular abnormalities in various types of blood vessels, including both microvessels and large vessels, under diabetic conditions. Traditional Chinese medicine (TCM) possesses the characteristics of “multiple components, multiple targets and multiple pathways,” and its safety has been demonstrated, particularly with minimal toxicity in liver and kidney. Thus, TCM has gained increasing attention from researchers. Moreover, recent studies have indicated that Chinese herbal medicine and its active compounds can improve vascular damage in diabetes by regulating autophagy. Based on this background, this review summarizes the classification, occurrence process, and related molecular mechanisms of autophagy, with a focus on discussing the role of autophagy in diabetic vascular damage and the protective effects of TCM and its active compounds through the regulation of autophagy in diabetes. Moreover, we systematically elucidate the autophagic mechanisms by which TCM formulations, individual herbal extracts, and active compounds regulate diabetic vascular damage, thereby providing new candidate drugs for clinical treatment of vascular complications in diabetes. Therefore, further exploration of TCM and its active compounds with autophagy-regulating effects holds significant research value for achieving targeted therapeutic approaches for diabetic vascular complications.

## 1 Introduction

Diabetes is a chronic disease characterized by metabolic disorders, primarily involving disturbances in the metabolism of glucose and lipid, and is accompanied by various chronic complications. With the development of global economy and changes in people’s lifestyles, the incidence of diabetes has been increasing year by year. According to statistics, type 2 diabetes accounts for the majority of diabetes cases and is often accompanied by one or more chronic complications (Wang P. et al., 2022). Diabetic chronic complications refer to the chronic damage caused by long-term hyperglycemic conditions to tissues and organs in the body, mainly including cardiovascular diseases, diabetic nephropathy, neuropathy, retinopathy, and non-healing wounds. These complications are the main causes of disability and premature death in patients, all of which are originate from diabetic vascular complications (Wang C.-Y. et al., 2023). Due to its high prevalence, poor prognosis, and heavy burden on healthcare costs, diabetic vascular complications have become a major public health issue. Currently, the molecular and pathophysiological mechanisms underlying diabetes-induced vascular complications are still not fully elucidated. Although ome progress has been made in the clinical treatment of diabetic vascular complications (Chen, 2018), such as controlling blood glucose, lipids, blood pressure, and lifestyle changes, specific therapeutic methods are still lacking.

To date, an increasing number of studies have shown a certain relationship between autophagy and the progression of diabetic vascular complications (Wei et al., 2021). Therefore, in this review, we provide an overview of the latest research progress on autophagy in diabetic vascular complications. Notably, we also introduce various traditional Chinese medicines (TCMs) that improve symptoms and molecular mechanisms of diabetic vascular complications by regulating autophagic flux. Furthermore, TCMs with autophagy-regulating capabilities hold promising prospects as a treatment strategy for diabetic vascular complications.

## 2 Autophagy

### 2.1 Classification of autophagy

**FIGURE 1:**
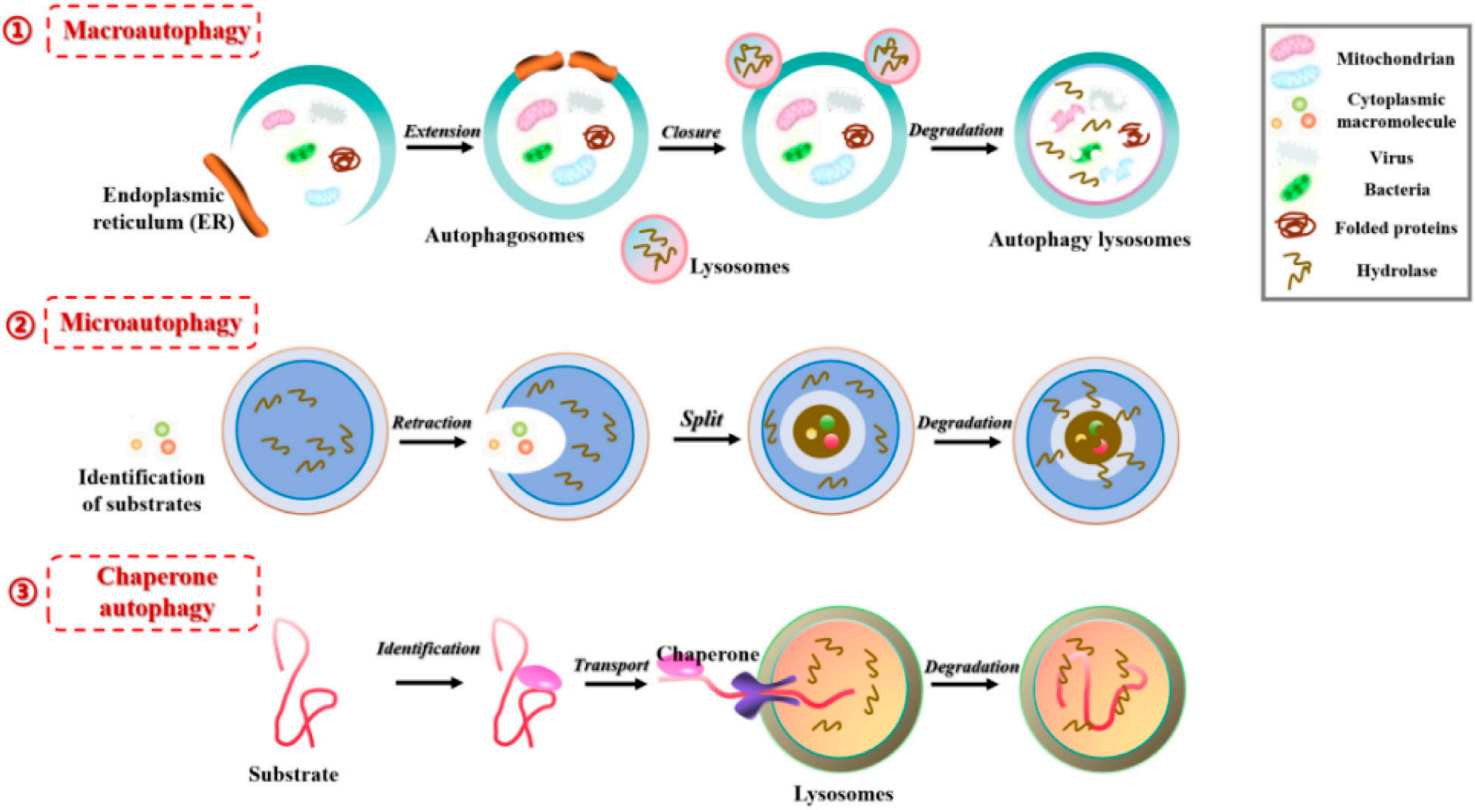
Three different forms of autophagy in mammals: 1) Macroautophagy, the gradual engulfment of cytoplasmic macromolecules and small organelles by autophagosomes, the autophagosomes are then delivered to lysosomes to form autolysosomes, where contents are degraded with the help of lysosomal hydrolytic enzymes; 2) Microautophagy, involves the direct invagination of lysosomal or vacuolar membranes to engulf cytoplasmic substances or organelles in a process of non-selective lysosomal degradation, without the involvement of autophagosomes; 3) Chaperone-mediated autophagy, involves the binding of molecular chaperone protein to specific soluble substrate protein molecules, followed by transport to lysosomes through the lysosome membrane receptor, and degraded by lysosomal hydrolytic enzymes.

Autophagy is a highly conserved pathway of lysosomal degradation that primarily involves the degradation of autophagosomes recruited to sequester misfolded proteins, damaged organelles, exogenous pathogens, and other substances by lysosomal hydrolytic enzymes. This process facilitates the clearance and recycling (degradation of contents to generate nutrients for cellular growth), thereby ensuring the homeostasis of the intracellular environment. Based on the occurrence process, autophagy can be classified into macroautophagy, microautophagy, and chaperone-mediated autophagy (Figure 1). Macroautophagy refers to the gradual engulfment of cytoplasmic macromolecules and small organelles by autophagosomes, which are double-membraned vesicles that form by extension and closure. The autophagosomes are then delivered to lysosomes to form autolysosomes, where contents are degraded with the help of lysosomal hydrolytic enzymes. Generally, autophagy refers to the macroautophagy. Microautophagy involves the direct invagination of lysosomal or vacuolar membranes to engulf cytoplasmic substances or organelles in a process of non-selective lysosomal degradation, without the involvement of autophagosomes. Chaperone-mediated autophagy (mainly in mammals) involves the binding of molecular chaperone protein Hsc70 to specific soluble substrate protein molecules with the KFERQ motif, followed by transport to lysosomes through the lysosome membrane receptor LAMP2A, and finally degradation by lysosomal hydrolytic enzymes (Parzych and Klionsky, 2014).

**FIGURE 2:**
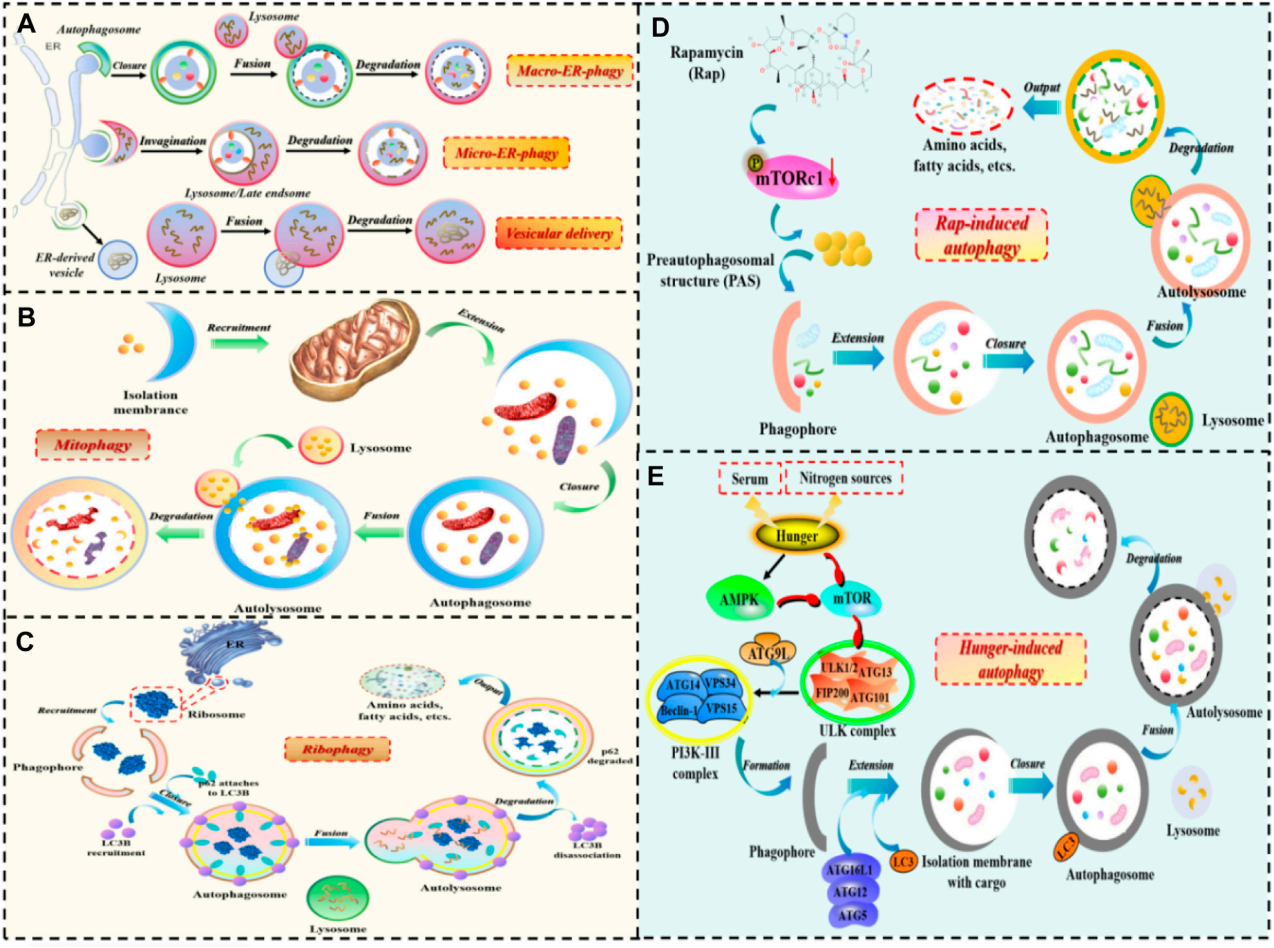
The occurrence process of selective autophagy and non-selective autophagy. 1) The process of selective autophagy: **(A)** ER-phagy; **(B)** Mitophagy; **(C)** Ribophagy. 2) The process of non-selective autophagy: **(D)** Autophagy induced by rapamycin; **(E)** Autophagy induced by starvation.

Notably, based on the selectivity of substrate degradation, autophagy can also be divided into selective autophagy and non-selective autophagy (Figure 2). Selective autophagy, as the name suggests, involves the involvement of selective autophagy receptors that directly bind to LC3 (Atg8 in yeast and plant cells), and then deliver specific degradation substrates to autophagosomes for degradation. Currently, types of selective autophagy include mitophagy, endoplasmic reticulum (ER)-phagy, ribophagy, pexophagy, and so on (Li W. et al., 2021; Vargas et al., 2023). Non-selective autophagy mainly refers to the relatively non-selective degradation of substrates. For example, autophagy induced by rapamycin, autophagy induced by nitrogen starvation, autophagy induced by serum starvation, and so on (Seglen et al., 1990).

### 2.2 The occurrence process of autophagy

The process of autophagy involves a series of consecutive stages, including initiation, nucleation, elongation, fusion, degradation, and recycling. These stages are highly regulated by autophagy-related genes (ATGs) and other auxiliary factors. In the initiation stage of autophagy, organelles such as mitochondria in the cytoplasm are first enclosed by vesicles called “isolation membranes”. These isolation membranes mainly originate from the ER and the Golgi apparatus. The vesicles eventually form double-membrane structures called autophagosomes, also known as initial autophagic vacuoles. Autophagosomes fuse with endosomes to form intermediate autophagic vacuoles. Various stimuli, such as oxygen deprivation and nutrient deficiency, induce the formation of autophagosomes, and this step involves two protein complexes: one is the Vps34 complex containing Vps34 (class III PI3K), Beclin1 (Atg6 in yeast), Atg14, and Vps15 (p150). The other is the ULK1 complex containing serine/threonine kinase ULK1 (Atg1 in yeast), which is an important positive regulatory factor in autophagosome formation. Subsequently, the elongation of autophagosomes leads to the formation of unique double-membrane structures called autolysosomes. This step requires the participation of two ubiquitin-like conjugation systems, both of which are catalyzed by Atg7: one system leads to the conjugation of Atg5-Atg12, which forms the Atg5-Atg12-Atg16L complex that can interact with the outer membrane of the extending phagophore. The other system involves the processing of Atg8, a protein encoded by the mammalian homolog of yeast Atg8, which leads to the generation of processed LC3. When autophagy is induced, LC3B is cleaved by the Atg4 protein to generate LC3-I, which is then activated by Atg7 and conjugated to phosphatidylethanolamine (PE) in the membrane to form processed LC3-II. Processed LC3-II is recruited to growing autophagosomes and its integration depends on Atg5-Atg12. Unlike Atg5-Atg12-Atg16L, LC3B-II is present on both the inner and outer surfaces of autophagosomes. The elongation and closure of autophagosomes both require the involvement of LC3B-II. After the closure of autophagosomes, the Atg16-Atg5-Atg12 complex dissociates from the vesicles, but a fraction of LC3-II remains covalently bound to the membrane. Therefore, LC3-II is commonly used as a marker to monitor the level of autophagy in cells. Once autophagosomes are fully closed to form autolysosomes, their outer membrane fuses with lysosomes to form autolysosomes. The fusion between autophagosomes and lysosomes requires the involvement of lysosomal membrane protein LAMP-1 and small GTPase Rab7. After fusion, the contents of the autophagocytic vesicles (including misfolded proteins, damaged organelles, invasive pathogens, etc.) are degraded by a series of acidic hydrolases in the lysozyme. The small molecules produced by degradation, especially amino acids, are transported back to the cytoplasm for protein synthesis and maintain normal cellular biological functions under starvation conditions (Yang and Klionsky, 2009; Glick et al., 2010; Devkota, 2017). After identification, in yeast, the main vacuolar amino acid effluxes during autophagy are Atg22 and other vacuolar permeases (such as Avt3 and Avt4), which help to understand the mechanism of nutrient cycling in the body, and these permeases also represent the final step of degradation and recycling (Abeliovich and Klionsky, 2001; Xu and Du, 2022). During the entire process of autophagy, both the cytoplasm and cell organelles are damaged, the most obvious being mitochondria and the endoplasmic reticulum (Qi and Chen, 2019; Wang S. et al., 2023). Although autophagy does not directly damage the cell membrane and cell nucleus, there is evidence that after initial fragmentation or digestion, the cell membrane and cell nucleus will eventually become lysosomes to digest and break down themselves, thus achieving a stable internal environment (Saftig and Puertollano, 2021).

### 2.3 Molecular mechanism of autophagy

Initially, autophagy research was mainly conducted in yeast. In the 1990s, Yoshimori and his team discovered multiple ATGs in the yeast model. They found that almost all yeast ATG homologues were found in higher eukaryotes. In 2003, *Klionsky* et al. named these genes ATG genes and studied the interactions between the encoded proteins and their autophagic functions. To date, researchers have identified more than 40 genes encoding ATG proteins in yeast, and in mammalian cells, about 20 core ATG genes regulate starvation-induced autophagy. These ATGs are constantly recruited near the vacuoles and assembled into autophagosome precursors. The classification and specific functions of these ATGs can be shown in Table 1. Subsequently, in April 2005, *Kliosnky* edited and published a new journal (“Autophagy”), leading to the increasing number of autophagy-related research papers indexed by PubMed (Ohsumi, 2014). Notably, on 3 October 2016, Japanese scientist Yoshinori Ohsumi was awarded the Nobel Prize in Physiology or Medicine for his “discovery of the mechanism of autophagy”, and the autophagy research had since received widespread attention from scholars in various fields (Długońska, 2017), with the molecular mechanisms of autophagy gradually being elucidated.

**TABLE 1:** The classification and function of ATGs.

Mammals	Yeast	Function
ULK1/2	ATG1	It is involved in initiation of autophagy, membrane targeting, membrane curvature sensing, and lipid vesicle tethering
ATG2A/B	ATG2	It is important for ATG9 recruitment to expand autophagosome
ATG3	ATG3	It is involved in LC3 lipidation and maintaining mitochondrial homeostasis
ATG4A-D	ATG4	It is involved in LC3 activation and delipidation
ATG5	ATG5	It is involved in autophagosome formation/elongation and in LC3 activation
Beclin-1	ATG6	It is involved in lipid binding and membrane deformation
ATG7	ATG7	It is involved in LC3 and ATG12 conjugation, and forms a thioester bond with ATG8
MAP1LC3A-C, GABARAPs, GATE-16	ATG8	It is involved in modifier, used as autophagosome marker, recognizes the cargo-specific adaptors
ATG9L1/L2	ATG9	It is involved in phagophore expansion, and interacts with ATG2-WIPI complex
ATG10	ATG10	It is involved in the E2-like enzyme in ATG12 conjugation with ATG5
ATG12	ATG12	It is involved in modifier, forms an E3 complex with ATG5 and ATG16, and interacts with ATG3
ATG13	ATG13	It is involved in initiation of autophagy, targets mTOR signaling pathway, interacts with ATG1, bridges ATG1 and ATG17-ATG31-ATG29, binds to LC3, and interacts with ATG101
ATG14 (Barkor)	ATG14	It is subunit of VPS34-PI3K complex, interacts with Beclin-1 to assemble the autophagic-specific complex, targets membrane and senses membrane curvature, and promotes membrane fusion
ATG16L1/L2	ATG16	It binds to ATG5-ATG12 complex acting as part of the E3 enzyme complex
RB1CC1/FIP200	ATG17	It is involved in initiation of autophagy, interacts with ATG13 and ATG9, forms ternary complex with ATG31 and ATG29; and senses membrane curvature
WIPI1-4	ATG18	It is part of the ATG2-WIPI complex which is important to autophagosome; binds to PI3P; required for the retrograde transport of ATG9; and complexes with ATG2
ATG101	—	Interact with ATG13 and forms the ULK-ATG13-ATG101-FIP200 complex

**FIGURE 3:**
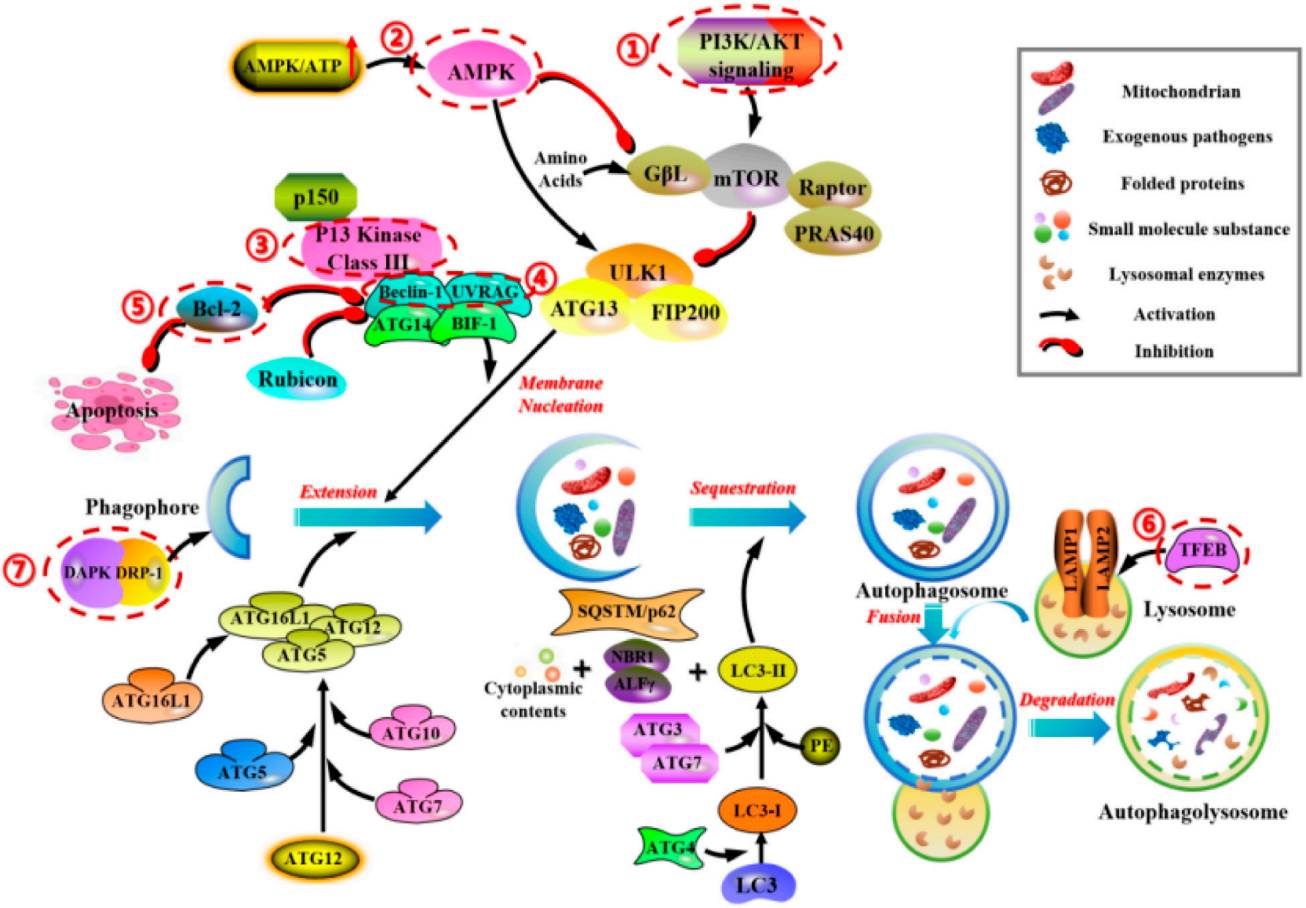
Main molecular mechanisms of autophagy in mammals. 1) PI3K/AKT/mTOR regulates the cell-cell signaling and transport of autophagic vesicles. 2) AMPK-TSC1/2-mTOR regulates the energetic stress signaling and transport of autophagic vesicles. 3) 3-methyladenine (3-MA) inhibits autophagy by suppressing the activity of Class III PI3K. 4) Beclin-1 and UVRAG, act as positive regulators, form the Class III PI3 complex to regulate autophagy. 5) Anti-apoptotic factor Bcl-2, acts as a negative regulator, form the Class III PI3 complex to regulate autophagy. 6) TFEB affects autophagic activity by interfering with the biological processes of lysosomes. 7) DAPK and DRP-1 can participate in the induction process of autophagy.

As a common physiological regulatory mechanism in mammalian cells, mTOR and AMPK are the two key molecules regulating autophagy activity in mammals, mainly involving the phosphatidylinositol 3-kinase (PI3K)-AKT-mTOR and AMPK-TSC1/2-mTOR signaling pathways. Additionally, other signaling pathways regulate autophagic activity to a certain extent. For example, 3-methyladenine (3-MA) inhibits autophagy by suppressing the activity of Class III PI3K, Transcription factor EB (TFEB) affects autophagic activity by interfering with the biological processes of lysosomes, Beclin-1 and UVRAG act as positive regulators, anti-apoptotic factor Bcl-2 acts as a negative regulator, and they together form the Class III PI3 complex to regulate autophagy. Death-associated protein kinase (DAPK) and DAPK-related protein kinase (DRP-1) can participate in the induction process of autophagy (Figure 3).

#### 2.3.1 PI3K-AKT-mTOR

There is a close relationship between the PI3K/AKT/mTOR signaling pathway and the cell autophagy response (Xu et al., 2020). Among them, PI3K can phosphorylate intracellular inositol lipids, regulate intercellular signaling and intracellular vesicle transport. According to their sequence homology and substrate specificity, they can be divided into three categories: Class I PI3K can produce 3-phosphoryl inositol lipids and directly activate signaling pathways. Class II and Class III PI3K regulate intracellular trafficking and autophagy pathway membrane transport, which have indirect effects on cell signaling. Notably, Class I PI3K is a heterodimer of the p110 catalytic subunit and the p85 regulatory subunit, which can be divided into p110α catalytic subunit and p110β catalytic subunit according to the coding gene. The roles of these two subunits in cell autophagy are also different. The p110α catalytic subunit plays an inhibitory role in cell autophagy through the NF-E2 P45-related factor 2-antioxidant response element-dependent pathway and activation of AKT protein. Conversely, the p110β catalytic subunit plays a promoting role in cell autophagy by binding to RAS-related protein 5 and activating forkhead box protein O (FoxO).

AKT is a downstream target of PI3K, which can inhibit the expression of ATG by inhibiting the activity of FoxO transcription factors. ROS level also downregulates the expression of FoxO protein, thereby inhibiting cell autophagy (Cheng, 2019). In addition, AKT not only regulates cell autophagy at the transcriptional level, but also participates in the regulation of several autophagy proteins. It is found that AKT had the ability to inhibit cell autophagy independently of mTORC1. Only when a mixture of rapamycin, PI3K and autophagy inhibitors are added with AKT inhibitors can be completely blocked cell autophagy. In the terms of site, AKT phosphorylates ULK1, a protein initiating cell autophagy at S774, which alters its conformation and regulates its intracellular location, resulting in its inactivation (Mohamed et al., 2021). AKT phosphorylates another autophagy protein Beclin-1 at S295, leading to its inactivation, thus inhibiting the formation process of Beclin-1-PI3K complex and blocking cell autophagy. Meanwhile, at the S295 site, using AKT inhibitors and up-regulating PTEN can also prevent Beclin-1 phosphorylation (Wang G. et al., 2021). Thus, AKT can inhibit cell autophagy by phosphorylating ULK1, Beclin-1 and other autophagy proteins to inactivate them.

mTOR can include two types: mTOR Complex 1 (mTORC1) and mTOR Complex 2 (mTORC2), both of which play important roles in cell autophagy (Kim and Guan, 2015). Among them, mTORC1 is mainly involved in the regulation of energy metabolism, cell growth, and the sensing of environmental changes. When the body is in an environment with sufficient oxygen, energy, and growth factors, the activity of mTORC1 can be activated, resulting in the upregulation of nucleotides, proteins, and fatty acid synthesis, and inhibiting cell autophagy, thereby promoting the accumulation of biomass and cell growth. In contrast, when the body is in an acidic, nutrient-deficient environment, the activity of mTORC1 can be inhibited, thus promoting the initiation of cell autophagy. mTORC2 is activated by growth factors and focuses on regulating the reconstruction of the cytoskeleton and cell survival. As a regulatory protein, both mTORC1 and mTORC2 can participate in the regulation of autophagy, and play essential roles in maintaining the dynamic balance between cell synthesis and degradation processes. Studies have found that mTORC1 mainly inhibits cell autophagy by phosphorylating proteins such as Unc-51-like kinase complex, ATG13, ATG14, protein nuclear receptor binding factor 2, and regulating the activity of transcription factors EB/E3, which are related to the formation and maturation of autophagosomes (Noda, 2017; Rabanal-Ruiz et al., 2017). In contrast, mTORC2 can inhibit cell autophagy by suppressing the protein kinase sgk1 and promoting autophagy by up-regulating the expression of mitochondrial outer membrane voltage-dependent anion channel protein 1 (Ballesteros-Álvarez and Andersen, 2021).

#### 2.3.2 AMPK-TSC1/2-mTOR

mTOR is a major regulatory molecule for cell growth and metabolism, promoting anabolic processes such as ribosome biogenesis and the synthesis of proteins, nucleotides, fatty acids, and lipids, while inhibiting catabolic processes such as autophagy. Dysregulation of mTOR signaling is often associated with numerous human diseases, including diabetes, neurodegenerative diseases, and cancer (Zhu et al., 2019b; Yang et al., 2019; Gremke et al., 2020). mTOR is a serine/threonine kinase belonging to the PI3K-related kinase (PIKK) family and can interact with several proteins to form two distinct complexes, known as mTORC1 and mTORC2. Numerous studies have shown that mTORC1 is a key regulator of autophagy, controlling different steps in the autophagic process (such as nucleation, autophagosome extension, maturation, and termination) (Martens, 2018). Therefore, mTORC1 is a promising target for regulating autophagy. In the activation process of mTORC1, it is often subjected to various stimuli (such as growth factors, nutrients, energy, and stress signals) and activated fundamental signaling pathways (such as PI3K, MAPK, and AMPK). Among them, the AMPK-TSC1/2-mTOR pathway is a classical autophagic pathway. Under conditions of abundant glucose, active mTORC1 inhibits autophagy initiation by phosphorylating specific sites (Ser 757) of ULK1 and disrupting the interaction between ULK1 and AMPK. In contrast, when glucose is scarce, AMPK is activated, leading to the phosphorylation of mTORC1 by AMPK, subsequent interaction between ULK1 and AMPK, and activation of ULK1 to initiate autophagy (Kim et al., 2011).

#### 2.3.3 Class III PI3K

PI3Ks is a class of kinases that plays a crucial role in cell signaling pathways, specifically catalyzing the phosphorylation of the 3-hydroxyl group of phosphatidylinositol (PtdIns). The catalytic products bind to specific domains on receptor proteins to mediate signal transduction, playing essential roles in processes such as cell growth, division, migration, vesicle trafficking, and autophagy (Farrell et al., 2013). PI3Ks can be divided into three classes (Type I, II, and III) based on their structural characteristics and substrates. Class III PI3K plays a key role in vesicle trafficking and autophagosome formation. Members of Class III PI3K and some of its complexes are highly conserved evolutionarily. In mammals, homologs of PI3KC3, p150, Beclin-1, ATG14L, and UVRAG are Vps34, Vps15, Vps30, Atg14, and Vps38, respectively. Notably, each member of the PI3KC3 complex can directly or indirectly regulate the activity of Vps34, thereby regulating autophagy. Vps34, as the third class of PI3 kinase in mammals (PIK3C3), phosphorylates phosphatidylinositol (PI) to produce phosphatidylinositol triphosphate (PI3P), which is crucial for the extension of autophagosome membranes and the recruitment of ATG proteins to autophagosomes. Vps34 is activated by binding to Vps15 and further associates with Beclin-1 to form the Vps34-Vps15-Beclin1 complex (Backer, 2008).

#### 2.3.4 TFEB

TFEB is a member of the basic helix-loop-helix leucine zipper transcription factor family MiT/TFE. Members of the MiT/TFE family play crucial roles in many cellular processes, particularly autophagy-lysosome biogenesis, cellular energy homeostasis, and metabolic processes. In vertebrates, the MiT/TFE transcription factor family consists of four evolutionarily conserved members: TFEB, MITF, TFEC, and TFE3. All MiT/TFE proteins, including TFEB, are evolutionarily conserved and can be found in primitive metazoans. TFEB is considered the primary regulator of lysosome biogenesis. TFEB can tightly link autophagy with lysosome biogenesis by regulating the expression of ATGs. Moreover, TFEB can also regulate lysosomal exocytosis. Increasing evidence suggests that dysregulation of TFEB activity leads to impaired autophagy-lysosome function, resulting in numerous human diseases, such as neurodegenerative diseases (including Alzheimer’s disease and Parkinson’s disease), lysosomal storage disorders, metabolic disorders, cancer and so on (He et al., 2019; Kim et al., 2021; Gu et al., 2022; Tan et al., 2022). Given the crucial role of TFEB in human diseases, regulating TFEB activity may represent a novel therapeutic target for clinical and regenerative applications. Studies have shown that the molecular mechanisms regulating TFEB are closely related to its subcellular localization (cytoplasmic and nuclear shuttling) and signal transduction (Willett et al., 2017). Autophagy is an adaptive response of cells to nutritional stress, and its activation is essential for maintaining homeostasis in the body. Initially, TFEB is mainly localized in the cytoplasm and can be transferred to the nucleus to cope with starvation. Upon re-feeding after hunger, TFEB’s re-localization can be restored. Moreover, the upstream molecules of TFEB (such as mTORC1) can also induce its nuclear localization after regulation. It is noteworthy that many stimuli (such as endoplasmic reticulum stress, infection, mitochondrial damage, and inflammation) can also promote TFEB’s nuclear localization. The activation and subcellular localization of TFEB are mainly controlled by protein interactions and post-translational modifications. Interestingly, the subcellular localization of TFEB is strictly controlled by phosphorylation. mTOR kinase is a known major kinase that phosphorylates TFEB (at sites S122, S142, and S211) and regulates its intracellular localization. Phosphorylation of TFEB at site S142 is also mediated by ERK2. Recent studies have also found that STUB1 regulates TFEB activity by targeting phosphorylated TFEB (S142 and S211) for ubiquitin-mediated proteasomal degradation (Sha et al., 2017).

#### 2.3.5 Beclin-1 and UVRAG

In yeast, Atg6/Vps30, which is a homolog of the mammalian Beclin-1 protein, is a protein involved in both autophagy and apoptosis pathways in the Vps34 complex. In mammals, Beclin-1 only participates in the autophagy process. In yeast, Atg6/Vps30 does not directly bind to Vps34 but forms a complex with Vps34 through Atg14 or Vps38, respectively participating in autophagy and protein sorting. Human Beclin-1 shares 24.4% homology with yeast Atg6/Vps30 and has been confirmed to play a crucial role only in the autophagy process, without participating in vesicle transport. Researchers have also analyzed the structure of Beclin-1, which contains a BH3 domain, making it a member of the Bcl-2 family and capable of binding to Bcl-2 (Cao and Klionsky, 2007). Furthermore, Beclin-1 contains an ECD domain (a key domain for binding of Beclin-1 to type III PI3K) and a CCD domain. Intermolecular interactions through the CCD domain allow Beclin-1 to form homodimers and heterodimers with UVRAG and Atg14 (Noble et al., 2008). Subsequently, the CCD domain of Beclin-1, a member of the Bcl-2 family, has been analyzed after studying its crystal structure. The results indicate that the surface a-d pairings and charge properties of key residues in the CCD domain directly affect the stability of dimers and the formation of different PI3KC3 complexes (Li et al., 2012). Therefore, Beclin-1 can bind to Bcl-2 and Bcl-XL to regulate apoptosis, as well as form complexes with type III PI3K to play a crucial role in autophagy, serving as an essential protein for communication and coordination between apoptotic and autophagic pathways.

UVRAG was initially discovered in 1990 by *Teitz* et al. while searching for genes responsible for UV damage resistance and DNA repair in cells, and the cDNA sequence of the protein was only cloned at that time. In 1997, Perelman et al. (1997) localized UVRAG to the 11q13 subregion of human chromosome 11, between D11S916 and D11S906, noting that the region is amplified in various cancers, suggesting a potential relationship between UVRAG and tumorigenesis. The study also analyzed the structure of UVRAG, indicating a CCD domain and a PEST domain, which may contribute to UVRAG’s metabolic instability. Ionov et al. (2004) identified two genes, UVRAG and p300, as likely causes of microsatellite instability in colorectal cancer, further confirming the close relationship between UVRAG and various diseases, including tumors. Subsequent research also demonstrated a correlation between UVRAG and microsatellite instability in gastric cancer (Kim et al., 2008). In addition to tumors, UVRAG deficiency can cause situs inversus in the thoracic and abdominal visceral (Iida et al., 2000; Goi et al., 2003). Current molecular biology research suggests that the CCD domain of UVRAG can bind to the CCD domain of Beclin-1 to form heterodimers, thereby stabilizing the Beclin-1-PI3KC3 complex and enhancing PI3KC3 kinase activity. This activation promotes autophagy as a positive regulatory factor. However, UVRAG deficiency may potentially induce cancer by downregulating autophagy (Zhong et al., 2009). Meanwhile, other studies have reported that UVRAG participates in autophagy through vesicle transport. UVRAG can form complexes with C-Vps, which function independently of the UVRAG-VPS34 complex. This complex primarily activates Rab7 GTPase activity, promoting fusion between membranous vesicles, including intracellular vesicle fusion and autophagosome fusion with late endosomes/lysosomes. This process accelerates the degradation of phagocytosed materials, promoting maturation of autophagosomes and the autophagic process (Liang et al., 2008). Therefore, the UVRAG-C-Vps complex can simultaneously promote both vesicle transport and maturation of autophagosomes.

#### 2.3.6 Anti-apoptotic factor Bcl-2

The Bcl-2 protein family can be divided into three subgroups: anti-apoptotic proteins, such as Bcl-2, Bcl-xL and Mcl-1, which contain the common BH1-BH4 structural domain. The pro-apoptotic proteins, such as Bax and Bak, which contain the common BH1-BH3 structural domain. And the pro-apoptotic proteins, such as Bad, Bid, Bim, Noxa, BNIP3 and Puma, which only contain the BH3 structural domain (Kale et al., 2018). Among them, Bcl-2 protein not only participates in cell apoptosis but also participates in cell autophagy and plays a key dual regulatory role in both (Liu et al., 2019b). At present, the classic theory for Bcl-2 regulation of autophagy and apoptosis is through the regulation of Beclin-1 (Xu and Qin, 2019). Beclin-1 is a mammalian autophagy gene homologous to the yeast ATG6/VSP30 autophagy gene and is the earliest discovered key factor in regulating autophagy. It is a BH3-only protein with three important structural domains: BH3, CCD, and ECD. Beclin-1 can bind to multiple proteins through these domains to form complexes that induce the localization of autophagy-related proteins to the autophagosome membrane and regulate the formation and maturation of autophagy. The proteins that make up this complex mainly include VPS34, UVRAG, Ambral, and Bcl-2. Among them, Bcl-2 contains the same BH3 structural domain as Beclin-1, and Bcl-2 can bind to Beclin-1 through this domain. When Bcl-2 and Beclin-1 form a complex, it can inhibit autophagy. When the classic apoptosis program is activated, activated Caspase-3 cleaves Beclin-1, thereby inhibiting autophagy. However, *Lindqvist LM* et al. have all found that the anti-apoptotic proteins represented by Bcl-2 do not directly bind to Beclin-1 to directly regulate cell autophagy, but indirectly regulate autophagy by inhibiting Bax/Bak-mediated apoptosis. That is, in the absence of Bax and Bak, Bcl-2 cannot significantly induce cell autophagic death (Lindqvist et al., 2014; Lindqvist and Vaux, 2014).

#### 2.3.7 DAPK and DRP-1

Recent studies had shown that the expression of death-associated protein kinase (DAPK) and DAPK-related protein kinase (DRP-1) could trigger two important non-caspase-dependent cytoplasmic events: 1) membrane vesicle growth (observed in any form of cell death). 2) proliferative autophagy (a typical characteristic of autophagy). And when autophagosomes were found to contain DRP-1, it indicated that this kinase directly participated in the process of autophagy (Inbal et al., 2002).

## 3 Autophagy in vascular lesions

Clinically, patients with diabetes often exhibit insulin resistance and hyperinsulinemia, which are commonly accompanied by hypertension, dyslipidemia, obesity, and other metabolic syndrome manifestations. Moreover, changes in abnormal vascular endothelial cells, coagulation, and platelet function in diabetes can lead to vascular damage, such as atherosclerosis, thereby causing a series of vascular lesions. Diabetic vascular lesions mainly include microvascular lesions and macrovascular lesions. Among them, microvascular lesions include retinopathy, peripheral neuropathy, and diabetic nephropathy, while macrovascular lesions include coronary heart disease and stroke. The symptoms produced by diabetic vascular lesions are mainly related to the location and severity of the lesioned vessels. The impact of diabetes on large vessels is mainly manifested as organ ischemia, such as chest stuffiness and chest pain caused by coronary heart disease, hemiplegia and aphasia caused by stroke, and cold feet, numbness, and claudication caused by peripheral arterial disease. When it accumulates to microvessels, it may lead to diabetic nephropathy (proteinuria and increased creatinine level in urine detected by auxiliary examination), retinopathy (blindness), peripheral neuropathy and microvascular disease (decreased sensation in extremity tips, ulcers, and infections). The basic treatment for diabetic vascular complications is to change unhealthy lifestyles while using medications to control blood sugar, hypertension, dyslipidemia, and obesity, which are risk factors for atherosclerosis. Additionally, it involves treating the damage of target organ corresponding to the vascular lesions. However, since diabetes simultaneously damages both large and microvessels throughout the body, the treatment difficulty lies in the fact that most patients have multiple vessels involved in the disease, making it difficult to restore blood supply to the target organs. Therefore, the treatment and prevention of diabetic vascular lesions remain a clinical challenge. Recent studies have increasingly shown that autophagy plays a crucial role in the development of diabetic vascular lesions, mainly involving vascular permeability, angiogenesis, vascular endothelial cell function, and vascular endothelial cell senescence (Salabei and Hill, 2013; Sanhueza-Olivares et al., 2022; Liu et al., 2023). Next, we will further elucidate the relationship between autophagy and these factors, laying the theoretical foundation for the application of autophagy-related preparations in diabetic vascular lesions and broadening the clinical treatment methods for this type of disease.

### 3.1 The relationship between autophagy and vascular permeability and neovascularization

Diabetic vascular lesions are mainly divided into microvascular lesions and macrovascular lesions. Endothelial cells located in the innermost layer of blood vessels are shaped like spindles or polygons, not only acting as a semi-permeable barrier between blood and vascular smooth muscle, but also serving as effector organs that sense and respond to changes in the external environment. They are important determinants of whole-body and local hemodynamic function and regulation of vascular smooth muscle cell proliferation. Therefore, endothelial cells play a key role in maintaining blood flow, regulating vascular permeability, and controlling the growth and proliferation of vascular smooth muscle cells.

Autophagy is an important metabolic process within cells, playing a crucial role in maintaining cellular homeostasis, clearing damaged proteins and organelles, regulating cell growth and death, and so on. In the terms of vascular permeability and neovascularization, autophagy also plays a certain role. On the one hand, autophagy can regulate vascular permeability. Studies have shown that autophagy plays an important role in regulating the function and stability of endothelial cells. Autophagy can clear harmful substances and damaged proteins in endothelial cells, and can maintain the stability of intracellular environment, so as to protect the function of endothelial cells. The loss of endothelial cell function will lead to the occurrence and development of atherosclerosis in diabetic patients. However, the mechanism of endothelial cell dysfunction in diabetic patients has not been fully understood. *Zhang YK* et al.'s research (Zhang et al., 2022) found that liraglutide, a glucagon-like peptide-1 (GLP-1) receptor agonist, acted on the upstream of the PINK1/Parkin pathway, effectively offset high glucose-induced cellular dysfunction by inhibiting the PINK1/Parkin-dependent mitochondrial autophagy. Therefore, it is very necessary to use liraglutide as an adjuvant treatment for type 2 diabetes to reduce the risk of atherosclerosis, and further research is needed to examine the exact molecules, including the SIRT1 upstream of the liraglutide targeted to maintain mitochondrial homeostasis PINK1/parkin pathway. In addition, autophagy can also regulate vascular permeability via adjusting the apoptosis and inflammatory response of endothelial cells. As shown by *Zheng YZ* et al.'s research (Zheng et al., 2022), melatonin directly reduced the expression of endothelial cell injury markers in HCAECs, thereby alleviating the vascular inflammation induced by *candida* albicans water-soluble components (CAWS) in a Kawasaki disease mouse model. Mechanistically, melatonin promotes autophagy by activating the melatonin/melatonin receptor (MT)/cAMP response element binding protein (CREB) pathway and upregulating AGT3 expression, so as to inhibit cell apoptosis in an autophagy-dependent manner. Moreover, melatonin also reduced the production of pro-inflammatory cytokines in macrophages and indirectly reduced immunopathological damage to HCAECs. This study shows that melatonin protects endothelial cells in Kawasaki disease by inhibiting cell apoptosis in an autophagy-dependent manner and reducing immunopathological damage mediated by macrophages. On the other hand, autophagy also plays a vital role in angiogenesis. Angiogenesis refers to the formation of new blood vessels, which plays a key role in tissue repair and regeneration. Research has found that autophagy can promote the proliferation and migration of vascular endothelial cells, participating in the process of angiogenesis. Autophagy affects the occurrence and development of angiogenesis by regulating vascular endothelial cell growth factors and signaling pathways. 1α, 25-Dihydroxyvitamin D3 (1,25D) is a hormone form of vitamin D, which has been proven to be beneficial for vascular diseases. As Xiong et al. (2021) found that 1,25D could partially improve the impairment of cell proliferation and migration induced by AGEs in endothelial cells via using CCK8 assay and Trans-well assay. Through tube formation assay, Western blot, and RT-PCR assay, they demonstrated that AGEs impaired angiogenic capacity, which was closely related to the expression changes of angiogenesis-related genes (including VEGFA, VEGFB, VEGFR1, VEGFR2, TGFβ1, MMP2, and MMP9) in endothelial cells. 1,25D could promote the expression of these angiogenic and angiogenic markers. Using DCFH-DA, ELISA, and Western blot assays, they also found that AGEs-induced oxidative stress damaged the angiogenic capacity of endothelial cells, and 1,25D relieved the vascular dysfunction by inhibiting oxidative stress. Notably, excessive autophagy induced by AGEs damaged angiogenesis, which was regulated by 1,25D and AGEs through the PI3K/Akt signaling pathway. This study suggests that AGEs-induced oxidative stress and autophagy can lead to angiogenic disorders, and 1,25D can improve angiogenesis by inhibiting excessive autophagy and oxidative stress, providing new insights for the treatment of diabetic vascular complications.

All in all, the role of autophagy in vascular permeability and angiogenesis is multifaceted. Autophagy affects the regulation of vascular permeability by regulating the function and stability of vascular endothelial cells. Meanwhile, autophagy also participates in the process of angiogenesis, promoting the formation of new blood vessels. However, further research is needed to reveal the specific mechanisms and regulatory processes.

### 3.2 The relationship between autophagy and vascular endothelial cell function

Studies had confirmed that long-term hyperglycemic conditions could lead to vascular endothelial dysfunction in the body, and the vascular abnormalities in diabetes patients were closely related to the injury of hyperglycemia-induced vascular endothelial cells (Li F. et al., 2023). In addition, endothelial cell dysfunction could also limit the repair function of endothelial cells and decrease the angiogenic capacity (Eelen et al., 2015; Gimbrone and Garcia-Cardena, 2016), which were considered important pathogenic factors for the development of diabetic cardiovascular diseases.

Autophagy plays an important role in the functional disturbance of endothelial cells. When the function of endothelial cells is disturbed, it may lead to the occurrence and development of vascular diseases. The role of autophagy in the functional disturbance of endothelial cells is mainly reflected in the following aspects: (1) Regulating oxidative stress in endothelial cells: Oxidative stress is one of the important factors leading to functional disturbance of endothelial cells. Autophagy can reduce the degree of oxidative stress in endothelial cells by removing harmful substances and damaged proteins generated by oxidative stress, thereby protecting the cells. As shown in the research by Zhu et al. (2019a), exposure of human umbilical vein endothelial cells (HUVECs) to oxidized low-density lipoprotein (ox-LDL) reduced cell viability, increased the content level of malondialdehyde (MDA), and reduced the content level of superoxide dismutase (SOD) in a concentration-dependent manner. Pre-treatment with salidroside significantly enhanced cell viability and reduced lactate dehydrogenase (LDH) release in HUVECs exposed to ox-LDL. Further research also found that ox-LDL could induce autophagy in HUVECs, and salidroside could further enhance autophagy after pre-treatment. However, salidroside weakened the increase of oxidative stress induced by ox-LDL in HUVECs. Interestingly, ox-LDL reduced the expression levels of SIRT1 and FOXO1 mRNA and protein, and these changes could be reversed by salidroside. This suggests that salidroside has a protective effect on endothelial cells induced by ox-LDL, and the mechanism may be related to increasing SIRT1 and FoxO1 expression to induce autophagy. (2) Maintaining homeostasis of endothelial cells: Autophagy can remove harmful substances and aging proteins in endothelial cells, maintaining stability of the intracellular environment. This helps to protect the function and stability of endothelial cells, reducing the occurrence of functional disturbance. Studies have shown that endothelial cell dysfunction plays a fundamental role in the pathogenesis of atherosclerosis, and autophagy has a protective effect on the occurrence of atherosclerosis. As Zhang G. et al. (2021) studied the effect of oxLDL/β2GPI/anti-β2GPI complex on autophagy in vascular endothelial cells and explored its potential mechanism. They used HUVECs and mouse brain endothelial cell line (bEnd.3) as the models of vascular endothelial cells. The expression of autophagy protein was detected by Western blot analysis, autophagosome accumulation was observed by TEM, and autophagic flux was detected by RFP-GFP-LC3 adenovirus transfection and chloroquine treatment to inhibit lysosomes, so as to assess the level of autophagy. Western blotting was used to detect the expression of phosphorylated PI3K, phosphorylated AKT, phosphorylated mTOR, and phosphorylated eNOS. 3-Methyladenine (3-MA) and rapamycin were used to assess the role of autophagy in the impairment of endothelial cell function induced by oxLDL/β2GPI/anti-β2GPI complexes. Their results showed that oxLDL/β2GPI/anti-β2GPI complexes inhibited the endothelial autophagy through the PI3K/AKT/mTOR and eNOS signaling pathways and further promoted the endothelial cell dysfunction. The findings of this study provide a new mechanism for vascular endothelial damage in patients with atherosclerosis complicated by antiphospholipid syndrome (APS). (3) Regulating inflammation response in vascular endothelial cells: Inflammation response is one of the common characteristics of vascular endothelial cell dysfunction. Autophagy can inhibit the inflammation response of vascular endothelial cells by regulating inflammation-related signaling pathways, thus reducing the occurrence of functional disorders. Caveolin-1 (Cav-1) expression plays a related role in the formation of atherosclerosis by controlling NO production, vascular inflammation, low-density lipoprotein (LDL) uptake, and extracellular matrix remodeling. As Zhang et al. (2020) studied how Cav-1 expression in aortic endothelium affects autophagy and whether enhanced autophagy is beneficial to the atherosclerosis-protective phenotype observed in Cav-1-deficient mice. Their results showed that Cav-1 deficiency promoted autophagy activation in susceptible areas of aortic endothelium by enhancing autophagic flux. Mechanistically, they found that Cav-1 interacted with the ATG5-ATG12 complex and affected the cellular localization of autophagosome components in lipid rafts, thereby controlling autophagosome formation and autophagic flux. Pharmacological inhibition of autophagy weakened the atherosclerosis protection observed in Cav-1 mice by increasing endothelial inflammation and macrophage recruitment, thus identifying a new molecular mechanism for preventing atherosclerosis progression by Cav-1 deficiency. This study demonstrates that Cav-1 is a relevant regulator of aortic endothelial autophagy and suggests that pharmacological inhibition of Cav-1-activated autophagy may provide a potential therapeutic strategy for cardiovascular diseases including atherosclerosis. (4) Regulating apoptosis and proliferation in vascular endothelial cells: Imbalance in apoptosis and proliferation of vascular endothelial cells is also an important factor in functional disorders. Autophagy can maintain normal growth and death of vascular endothelial cells by regulating cell apoptosis and proliferation-related signaling pathways, thus reducing the occurrence of functional disorders. ANGPTL4 is a member of the angiopoietin-like protein family and participates in the regulation of angiogenesis, lipid metabolism, glucose metabolism, and redox reactions. As Zhan et al. (2022) found that over-expression of ANGPTL4 in HUVECs enhanced cell proliferation and clonal formation abilities *in vitro*, whereas knockdown of ANGPTL4 had the opposite effect. Moreover, ANGPTL4 expression was upregulated in HUVECs treated with palmitic acid (PA), and over-expression of ANGPTL4 prevented PA-induced endothelial damage. In their exploration of the mechanism, they demonstrated that ANGPTL4 regulated endothelial cell proliferation and apoptosis by modulating autophagy. Additionally, they found that ANGPTL4 levels were downregulated in atherosclerosis mouse serum and were closely related to autophagy-related proteins in atherosclerosis mouse aortic tissue. This study suggests that ANGPTL4 promotes endothelial cell proliferation and inhibits PA-induced endothelial cell damage, thereby preventing the development of atherosclerosis. Furthermore, Fan et al. (2023) evaluated the effects of Homoplantaginin (Hom) on vascular endothelial cells by using high-glucose-treated HUVECs and db/db mice. *In vitro*, Hom significantly inhibited cell apoptosis, promoted autophagosome formation and lysosome function, such as lysosome membrane permeability and expression of LAMP1 and cathepsin B. Autophagy inhibitors, such as chloroquine or bafilomycin A1, reversed Hom’s anti-apoptotic effects. Moreover, Hom promoted gene expression and nuclear translocation of TFEB. Knockdown of TFEB weakened the up-regulating effects of Hom on lysosome function and autophagy. Hom activated AMPK protein and inhibited the phosphorylation of mTOR, p70S6K, and TFEB proteins. AMPK inhibitors attenuated these effects, and molecular docking suggested a good interaction between Hom and AMPK proteins. Animal studies showed that Hom effectively upregulated the expression of p-AMPK and TFEB proteins, enhanced autophagy, reduced cell apoptosis, and alleviated vascular damage. These findings indicate that Hom can enhance autophagy through the AMPK/mTORC1/TFEB pathway, so as to improve the high glucose-mediated vascular endothelial cell apoptosis.

All in all, autophagy plays a crucial role in vascular endothelial cell dysfunction. Autophagy can protect vascular endothelial cell function and stability by regulating oxidative stress, maintaining cell homeostasis, inhibiting inflammatory responses, and regulating cell apoptosis and proliferation. Therefore, further study of the regulatory mechanisms of autophagy in vascular endothelial cell dysfunction has a great significance, which will help develop new therapeutic strategies to prevent the occurrence of vascular diseases.

### 3.3 The relationship between autophagy and vascular endothelial senescence

Increasing evidence suggests that persistent hyperglycemia is an important cause of vascular endothelial cell senescence in patients with diabetes (Li et al., 2019; Wang G. et al., 2021; Wan et al., 2022). In the normal physiological conditions, vascular endothelial cells are in a quiescent phase with a low rate of self-renewal. However, exogenous damaging factors (such as high glucose stimulation) can lead cells into the division phase, during which telomeres shorten continuously, resulting in senescence. The main characteristics of senescence include cell enlargement, enhanced SA-β-gal activity, cell cycle arrest, increased expression of senescence-associated genes and proteins, and increased cell inflammatory secretions. In addition, endothelial cell senescence plays a crucial role in the development of vascular aging and related cardiovascular diseases. Atherosclerotic lesions are often accompanied by endothelial cell senescence. As discovered by Brodsky et al. (2004), vascular endothelial cell senescence was closely related to the severity of vascular lesions in Zucker diabetic rats. Ebselen, as a potent nitrosative stress scavenger, could reduce protein tyrosine nitration modification and delayed vascular endothelial cell senescence, thus alleviating diabetic macrovascular lesions. Moreover, endothelial cell senescence causes decreased vascular elasticity and compliance, serving as one of the major causes of hypertension. Thus, endothelial cell senescence was closely related to diabetic macroangiopathy.

Autophagy plays a vital role in vascular endothelial senescence. Vascular endothelial cells are the inner layer of the vascular wall and play a key role in maintaining vascular function and stability. Following with age, vascular endothelial cells undergo senescence, which may lead to decreased vascular function and the development of related diseases. The role of autophagy in vascular endothelial senescence mainly includes: 1) Clearance of senescent cells and damaged materials: Autophagy can clear senescent vascular endothelial cells, including aged proteins and organelles. This helps to maintain the function and stability of vascular endothelial cells, reducing the negative impact of senescent cells on the surrounding environment. As discovered by Chen et al. (2014), angiotensin-converting enzyme inhibitors (ACEIs) or angiotensin II type 1 receptor blockers (ARBs) had beneficial effects on high glucose-induced endothelial responses. High glucose activation of the RAS enhanced mitochondrial damage and increased senescence, apoptosis, and autophagic responses in endothelial cells, which were simulated by using angiotensin (Ang) II. However, the negative effects of hyperglycemia were inhibited by ACEI or ARB. Direct mitochondrial damage caused by 3-chloro-benzoyl cyanide (CCCP) resulted in similar adverse effects to high glucose or Ang, and eliminated the protective effects of ACEI or ARB. By impairing autophagy, high glucose-induced senescence and cell apoptosis were accelerated, and the beneficial effects mediated by ACEI or ARB were abolished. In addition, increased FragEL™ DNA fragmentation (TUNEL) positive cells, β-galactosidase activation, and expression of autophagy biomarkers were observed in diabetic patients and rats, which were reduced by ACEI or ARB treatment. These data indicate that autophagy prevents vascular endothelial cell senescence and apoptosis in high glucose-induced endothelial cells through RAS-mitochondrial regulation. 2) Maintenance of vascular endothelial cell function: Autophagy can protect the biological function of vascular endothelial cells by clearing harmful substances and maintaining stability within the cell. Specifically, autophagy can eliminate damaged proteins and organelles in senescent cells, reducing toxic substance accumulation within cells and maintaining normal cell function. Diabetic vascular complications are usually defined by vascular endothelial lesions. However, whether endothelial cells can switch from a quiescent state to a highly active state under high glucose stimulation, hindering vascular stability, and whether this process is related to diabetic vascular complications remain unclear. Survivin had been identified as an anti-apoptotic protein in tumors or epithelial cells, promoting proliferation or inhibiting cell apoptosis. Therefore, Xu et al. (2018) investigated the effects of high glucose on endothelial cell function and the potential involvement of survivin. They found that high glucose did not cause endothelial damage, but significantly promoted vascular endothelial cell proliferation and angiogenic capacity, indicating endothelial cell dysfunction characterized by a bias towards high activity. Simultaneously, upregulation of survivin was detected along with increased key components of the autophagic pathway, including LC3, Beclin-1, and p62. YM155 was a specific survivin inhibitor that could eliminate high glucose-induced endothelial hyperactivation. The application of autophagy inhibitor (3 MA) and agonist (rapamycin) supported the idea that survivin could act as a downstream effect or autophagy. These data suggest that the survivin/autophagy axis is a potential therapeutic target for diabetic vascular complications. 3) Regulating inflammatory response of vascular endothelial cells: Aging vascular endothelial cells often produce more inflammatory factors, leading to increased inflammation. Autophagy can inhibit the inflammatory response of vascular endothelial cells and reduce the occurrence of vascular endothelial senescence by regulating inflammation-related signaling pathways. Studies have shown that neutrophil migration from the circulation to infected or injured sites is a crucial immune response, which requires the disruption of endothelial cells on the inner side of blood vessels. Unregulated neutrophil transendothelial migration is pathogenic, but its physiological termination molecular basis is still unknown. As Reglero-Real et al. (2021) demonstrated that venular endothelial cells in inflamed tissues could exhibit strong autophagic responses, which are time-consistent with the peak of neutrophil transport and strictly localized to endothelial cell contacts. Meanwhile, in a mouse inflammation model, genetic ablation of autophagy in endothelial cells led to excessive neutrophil TEM and uncontrolled leukocyte migration, while pharmacological induction of autophagy inhibited neutrophil infiltration into tissues. Mechanistically, autophagy regulated the remodeling of endothelial cell junctions and the expression of key endothelial cell adhesion molecules, thus promoting intracellular transport and degradation. This study suggests that autophagy is an important regulator of leukocyte transport mechanisms in endothelial cells aimed at terminating physiological inflammation. Diabetic retinopathy (DR) is a chronic, progressive, and potentially harmful retinal disease associated with persistent hyperglycemia. Yu et al. (2023) established a DR model using high glucose-treated human retinal microvascular endothelial cells (HRMECs) and stachydrine (STA, a water-soluble alkaloid extracted from Leonurus heterophyllus) treatment in rats. They used corresponding kits to determine levels of ROS and inflammatory factors (including TNF-α, IL-1β, and IL-6), CCK-8 and EDU staining assays to analyze cellular growth, and immunofluorescence and Western blot techniques to detect the expression levels of LC3B-II, p62, p-AMPKα, AMPKα, and SIRT1 proteins. The results showed that STA decreased ROS and inflammatory factor levels in HRMECs treated with high glucose. Furthermore, after STA treatment, the protein levels of Beclin-1, LC3B-II, p-AMPKα, and SIRT1 increased in HRMECs treated with high glucose and STA-treated rat retinal tissues, while the level of p62 protein decreased. This study indicates that STA can effectively alleviate inflammation and promote DR progression via activating the AMPK/SIRT1 signaling pathway *in vitro* and *in vivo*.

All in all, autophagy plays an important role in endothelial aging within blood vessels. Autophagy can maintain the function and stability of endothelial cells by clearing aged cells and damaged substances, as well as regulating inflammatory responses, thereby maintaining the health state of endothelial cells. Therefore, further research on the regulatory mechanism of autophagy in endothelial cell aging may help develop new therapeutic strategies to delay vessel aging and related diseases.

## 4 Active compounds from Chinese herbs relieve diabetic vasculopathy by regulating autophagy

In recent years, more and more scholars have pointed out that Chinese herbs have a feature of “multi-component, multi-target, multi-pathway”, which not only conforms to the holistic thinking in traditional Chinese medicine but also fits the complex and changeable process of diabetes and its complications. In addition, according to the research of modern pharmacology, Chinese herbs and their active compounds not only have broad biological activities (such as antioxidant, anti-inflammatory, and anti-tumor) (Wang et al., 2013; Luo et al., 2019; Zhang Q. et al., 2021), but these substances have also been proven to be relatively safe, especially with less toxicity to liver and kidney (Yang et al., 2018; Liu et al., 2020; Zulkifli et al., 2023). Therefore, Chinese herbs (including single-flavor drugs and compound formulations) and their active substances are increasingly considered alternative sources for regulating patients with diabetes and its complications (Table 2–4).

**TABLE 2:** The active compound counteracts the effects of diabetic vascular damage by modulating autophagic flux.

Herbal extracts	Categories	Autophagy-related targets	*In vitro/In vivo*	Model	References
Salvianolic acid B	*Salvia miltiorrhiza Bge*	Beclin-1, Parkin, Pink1	*In vitro and vivo*	HG-induced HUVECs and db/db mice	Xiang et al. (2022)
Notoginsenoside R1	*Panax notoginseng (Burkill) F. H. Chen ex C. H. Chow*	LC3B, P62/SQTM1, Parkin, Pink1	*In vitro and vivo*	HG-induced rMC-1 and db/db mice	Zhou et al. (2019)
Notoginsenoside Fc	*Panax notoginseng (Burkill) F. H. Chen ex C. H. Chow*	LC3B, Beclin-1, P62/SQTM1	*In vitro and vivo*	Sprague-Dawley rat model induced by STZ and RAOECs induced by HG	Liu et al. (2019a)
Glycyrrhizic acid	Glycyrrhiza uralensis Fisch	LC3B, Beclin-1, P62/SQTM1	*In vivo*	Rat model of type I diabetes	Asseri et al. (2022)
Scutellarin	Scutellaria baicalensis Georgi	LC3B, Beclin-1, P62/SQTM1, ATG5, Parkin, Pink1	*In vitro*	HG-induced HUVECs	Xi et al. (2021)
Epigallocatechin gallate	*Green Tea*	LC3B	*In vitro*	Palmitic acid-induced BAEC	Kim et al. (2013)
Homoplantaginin	*Salvia japonica Thunb*	LAMP1, Cathepsin B, AMPK, mTOR, TFEB	*In vitro and vivo*	HG-induced HUVECs and db/db mice	Fan et al. (2023)
Resveratrol	*Aloe vera (L.) Burm. f*	LC3B, Beclin-1	*In vitro*	Gly-LDL-induced HUVECs	Sha et al. (2021)
Artesunate	*Artemisia caruifolia Buch.-Ham. ex Roxb*	LC3B, Beclin-1, P62/SQTM1, AMPK, SIRT1	*In vivo*	DR rat model induced by STZ	Li et al. (2023b)
Naringin	*Citrus reticulata Blanco*	mTOR, LC3B, Beclin-1, P62/SQTM1	*In vitro*	HG/HF-induced HUVECs	Wang et al. (2020a)
Geranylgeranylacetone	*Bayberry*	mTOR, LC3B	*In vivo*	Rat retinal I/R model	Zhang et al. (2023a)
Icariin	*Epimedium brevicornu Maxim*	mTOR, LC3B, P62/SQTM1	*In vitro*	H_2_O_2_-induced BM-EPCs	Tang et al. (2015)
Gypenoside XVII	*Gynostemma pentaphyllum (Thunb.) Makino*	ATG5, Beclin-1, LC3B, P62/sqtm1	*In vivo*	db/db mice	Luo et al. (2021)
Lentinan mushroom	*Lentinus edodes*	Beclin-1	*In vitro*	HG-induced HUVECs	Yang et al. (2022)

**TABLE 3:** A single herb counteracts the effects of diabetic vascular damage by modulating autophagy flux.

Single herbs	Autophagy-related targets	*In vitro/In vivo*	Model	References
Ginger	Beclin-1, Parkin, Pink1	*In vitro, In vivo*	HG-induced HUVECs and db/db mice	Li et al. (2021a)
Ginkgo Biloba Leaf Extract	mTOR, P62/SQTM1	*In vivo*	ApoE−/− mice model induced by STZ and high-fat diet	Tian et al. (2019)
Large-leaf yellow tea	LC3B, Beclin-1, ATG5, P62/SQTM1	*In vivo*	Diabetic mice and HG-induced HUVECs	Wang et al. (2022b)
Ginseng-Sanqi-Chuanxiong (GSC) extracts	LC3B, Beclin-1, P62/SQTM1, Parkin, Pink1	*In vitro*	HG/PA-induced HAEC	Wang et al. (2020b)
Aqueous anise extract	Beclin-1	*In vivo*	Rat model induced by STZ	Faried and El-Mehi (2020)
Ginseng lotus extract	LC3B, P62	*In vivo*	Ligate and reperfusion of the left anterior descending artery of rat	Li (2022)

**TABLE 4:** TCM compounds counteract the effects of diabetic vascular injury by regulating autophagic flux.

Chinese herbal compound preparations	Herbal composition	Autophagy-related targets	*In vitro/In vivo*	Model	References
Yigan Mingmu decoction	*Bupleurum, angelica, white peony, ligusticum wallichii, salvia miltiorrhiza, poria cocos, plantain seed, flos Buddlejae, tribulus terrestris, rhizoma alismatis, motherwort fruit, cassia seed*	LC3B	*In vivo*	SD rat model induced by STZ	Wang (2021)
Danzhi Jiangtang Capsules	*Radix pseudostellariae, rehmannia glutinosa, Peony bark, rhizoma alismatis, dodder, leech*	Beclin-1, ATG5, LC3B	*In vivo*	GK lower limb ischemic rats	Ni et al. (2018)
Huoxue Jiedu Jiangtang decoction	*Ginseng, ophiopogon japonicus, schisandra chinensis, cornus officinalis, birthplace, rhizoma dioscoreae, rheum officinale, turtle shell, peach kernel, peony bark, coptis chinensis*	LC3B, Beclin-1	*In vivo*	Rat model induced by STZ and high-fat diet	Fu et al. (2023)
Mingmu Xiaomeng tablets	*Pearl powder, grape, zinc acid*	LC3B, P62/SQTM1	*In vivo*	Rat model induced by STZ	Fang (2020)
Huangqi Gegen Decoction Combined with Gualou Decoction	Astragalus membranaceus, pueraria lobata, fructus trichosanthis, myrrh, licorice	Beclin-1	*In vivo*	SD rat model induced by STZ and high-fat diet	Bi, (2023)
Buyang Huanwu Decoction	*Astragalus membranaceus, angelica sinensis tail, red peony, earthworm, ligusticum wallichii, peach kernel, safflower*	LC3B, Beclin-1	*In vitro*	HG-induced HRCECs	Shi et al. (2022)

### 4.1 Compound Chinese herbal formulas alleviate diabetic vasculopathy by regulating autophagy

Yigan Mingmu decoction has the functions of soothing the liver and improving eyesight, as well as promoting blood circulation and diuresis. It is a Chinese herbal compound formulation that can improve retinal lesions in diabetic patients. Some researchers have confirmed through *in vivo* and *in vitro* experiments that Yigan Mingmu decoction can reduce the relative expression of TXNIP on Müller cells in the retina, keeping its autophagic level in balance, so as to damage mitochondria produced by oxidative stress can be eliminated through complete autophagy pathways, thus avoiding the release of inflammatory factors and reducing apoptosis and necrosis of cells, thereby exerting protective effects on the retina (Wang S. et al., 2021).

Danzhi Jiangtang Capsules is a Chinese herbal compound formula that has been used at Anhui Provincial Hospital of TCM for decades. In previous studies, it was found that Danzhi Jiangtang Capsules could alleviate calcification of diabetic rat lower limb endothelial cells, and the mechanism was to downregulate the expression of β-catenin in the nucleus, reduce the synthesis of β-catenin protein, thereby inhibiting the transcription of downstream cell phenotype genes, so as to achieve the purpose of preventing endothelial cell vascular calcification (Ni et al., 2023). In addition, Danzhi Jiangtang Capsules could activate the VEGF/Akt/eNOS and Ang/Tie2 signaling pathways that are suppressed in diabetic vascular lesions, thereby promoting the dissociation of the basement membrane of blood vessels, preparing for the initiation of angiogenesis, and ultimately promoting the regeneration of lower limb blood vessels (Li, 2017; Ni et al., 2020). Notably, a recent study found that Danzhi Jiangtang Capsules could promote angiogenesis and treat diabetic lower extremity vascular lesions by inhibiting the expression level of genes related to the mTOR pathway, inducing the formation of autophagosomes, and activating autophagy. Meanwhile, based on the network pharmacology and the molecular docking technology, it was found that Danzhi Jiangtang Capsules had multiple components, multiple targets, and multiple pathways for treating diabetic complications. Among them, 12 key components (including quercetin, luteolin, kaempferol, crocetin, paeoniflorin, ursolic acid, isorhamnetin, β-sitosterol, jingniping glycoside, gardnerilin A, alexin, and hyperin) and 17 core targets (including AKT1, TNF, IL6, IL1B, VEGFA, TP53, CASP3, JUN, EGFR, STAT3, FN1, PPARG, MYC, MMP9, PTGS2, CXCL8, and CCL2) were enriched. Its mechanism of action might be related to inhibiting inflammation, reducing oxidative stress, regulating cell apoptosis, adjusting immune response, and autophagy regulation (Ni et al., 2018).

Interestingly, TCM formula not only has a certain effect on microcirculation vessels, but also has protective effects on large blood vessels, which are gradually being studied by scholars. Huoxue Jiedu Jiangtang decoction is a traditional Chinese herbal formula that can improve the large blood vessel lesions of diabetic patients. Previous studies had shown that Huoxue Jiedu Jiangtang decoction could reduce the inflammation response in diabetic rats, reduce aortic cell apoptosis, inhibit the coupling of endoplasmic reticulum stress and inflammation response, so as to improve the arterial damage caused by diabetes (Fu et al., 2019; Cao et al., 2020). Notably, recent research had shown that Huoxue Jiedu Jiangtang decoction could affect the development of diabetic atherosclerosis through the autophagy pathway. As Fu et al. (2023) used streptozotocin (STZ) abdominal injection and high-fat feeding to establish a diabetic model and used aortic balloon injury to establish an atherosclerosis model. Meanwhile, blood was taken to detect glycated hemoglobin and serum triglyceride (TG), low-density lipoprotein cholesterol (LDL-C) levels, H&E staining was used to observe the pathological morphology of the aorta, and transmission electron microscopy (TEM) was used to observe autophagy phenomena and ultrastructures in aortic cells. RT-PCR was used to detect the expression levels of IRE-1, TRAF2, and GRP78 mRNA in aortic tissue, and Western blot was used to detect the expression of Beclin1 and LC3II proteins in aortic tissue. This study showed that Huoxue Jiedu Jiangtang decoction could protect the aorta by inhibiting ER stress caused by diabetic metabolic disorders, regulating endoplasmic reticulum homeostasis, and inhibiting excessive autophagy of aortic endothelial cells.

### 4.2 Single-flavor drug alleviates diabetic vasculopathys by regulating autophagy

Ginger, as a common and widely used spice, is rich in various chemical components, including phenolic compounds, terpenes, polysaccharides, lipids, organic acids, and fibrils (Zhou et al., 2021). Recent studies had found that ginger could have a certain effect on the vascular system. On the one hand, using cell cultures or animal models had shown that the active ingredients in ginger could alleviate oxidative stress and inflammation, increase NO synthesis, inhibit the proliferation of vascular smooth muscle cells, promote cholesterol excretion by macrophages, inhibit angiogenesis, block voltage-dependent Ca^2+^ channels, and induce autophagy (Li C. et al., 2021). On the other hand, in some clinical trials, ginger had been shown to have beneficial effects on blood lipids, inflammatory cytokines, blood pressure, and platelet aggregation (Daniels et al., 2022). These studies pointed to the potential benefits of ginger and its components in the treatment of vascular-related diseases (including hypertension, coronary artery disease, diabetes and so on).

In addition, studies had shown that ginkgo biloba extract could restore autophagic flux through the mTOR signaling pathway to inhibit ER stress, thus reducing atherosclerosis in diabetic ApoE−/− mice induced by STZ (Tian et al., 2019).

High glucose-induced vascular endothelial cell injury plays an important role in the development of diabetic vascular complications. Studies had shown that yellow tea had a protective effect on vascular endothelial cells. As Wang et al. (2022) studied the effect of n-butanol fraction of Huoshan large-left yellow tea extract (HLYTE) on high glucose-induced vascular endothelial cell injury by using HUVECs and diabetic mice as research subjects. In this study, they found that the extract of HLYTE could upregulate autophagy through the AMPK/mTOR pathway and inhibit oxidative stress response, thereby protecting vascular endothelial cells from high glucose-induced injury.

### 4.3 Active compounds alleviate diabetic vasculopathys by regulating autophagy

There is growing evidence that active compounds abundant in TCM can alleviate diabetic vascular injury through autophagy pathway. Salvianolic acid B is a non-protein amino acid in *Salvia miltiorrhiza Bge.* With a wide range of biological activities. Recent studies had found that Salvianolic acid B could alleviate diabetic endothelial and mitochondrial dysfunction by down-regulating endothelial cell apoptosis and mitochondrial autophagy (including Beclin-1, Parkin, Pink1 targets), thereby improving diabetic-induced vascular complications (Xiang et al., 2022).

Notoginsenoside R1 is a novel saponin extracted from *Panax notoginseng (Burkill) F. H. Chen ex C. H. Chow* with pharmacological properties, including treating diabetic encephalopathy, improving microcirculation disorders, and so on. As Zhou et al. (2019) found that the retinal vascular degeneration, retinal thickness reduction, and retinal function impairment in db/db mice were significantly alleviated after 12 weeks of oral administration of notoginsenoside R1 (30 mg/kg). Meanwhile, pre-treatment with notoginsenoside R1 significantly inhibited apoptosis of retinal Müller cells (rMC-1) in high glucose-induced rats and db/db mice, and suppressed oxidative stress and inflammation by inhibiting VEGF expression and increasing PEDF expression. Moreover, pre-treatment with notoginsenoside R1 upregulated the levels of PINK3 and Parkin in high glucose-induced rMC-1 cells and db/db mouse retinal cells, increased the LC3-II/LC3-I ratio, downregulated the levels of p62/SQSTM1, and enhanced the co-localization of GFP-LC1 puncta and MitoTracker in rMC-3 cells. In summary, this study demonstrated that notoginsenoside R1 could improve diabetic retinopathy by activating mitochondrial autophagy through PINK1-dependent mechanism. Notoginsenoside Fc is another novel saponin isolated from the leaves of *Panax notoginseng (Burkill) F. H. Chen ex C. H. Chow*. Numerous pharmacological activities of notoginsenoside Fc, such as anti-oxidant, anti-inflammatory, and anti-tumor effects, have been confirmed by many scholars as folk medicine or dietary supplement (Huang and Tian, 2016; Gong and Zhuang, 2022; Niu et al., 2023). In the terms of blood vessels, notoginsenoside Fc not only effectively counteracts the aggregation of platelet, but also shows potential benefits for re-endothelialization (Liu et al., 2018; Zhang X.-F. et al., 2023). Notably, interventional therapies, such as percutaneous transluminal angioplasty and endovascular stent implantation, have been widely used for treating diabetic peripheral vascular complications. However, re-endothelialization is an essential process after vascular injury during interventional therapy, and hyperglycemia in diabetes plays a crucial role in disrupting endothelial layer integrity, leading to delayed re-endothelialization and excessive intimal proliferation. Interestingly, as Liu et al. (2019a) found that notoginsenoside Fc counld accelerate re-endothelialization after vascular injury in diabetic rats by promoting endothelial cell autophagy (including LC3B, Beclin-1, P62/SQTM1 targets), indicating that notoginsenoside Fc might have therapeutic benefits for early endothelial damage and restenosis after intervention for diabetes-related vascular diseases.

Glycyrrhizic acid is the main active component of TCM *Glycyrrhiza uralensis Fisch*. Previous studies had shown that glycyrrhizic acid had a wide range of pharmacological effects, such as anti-oxidation, anti-inflammation, anti-viral, anti-cancer, and so on (Wang and Du, 2016; Sun et al., 2019; Asseri et al., 2022; Zhang Y. et al., 2023). In addition, many pieces of evidence had confirmed that glycyrrhizic acid could have certain improvement effects on diabetes and its related complications. It was reported that diabetes complications could further affect many organs and systems, including oral tissues and salivary glands. Among them, salivary glands played a vital role in maintaining oral homeostasis, and the parotid gland, submandibular gland, and sublingual gland were the main salivary glands responsible for synthesizing saliva. During the development of diabetes, the salivary glands could be negatively affected, leading to reduce saliva secretion, periodontal tissue damage, and reduce salivary function, oral mucosal lesions. Notably, recent studies had shown that glycyrrhizic acid might improve diabetes-induced sublingual gland damage by regulating oxidative stress, autophagy (including LC3B, Beclin-1, P62/SQTM1 targets), and angiogenesis, so as to restore the homeostasis of oral environment (Asseri et al., 2022).

Scutellarin is one of the main active components of TCM *Scutellaria baicalensis Georgi*. It has been reported that scutellarin has been used to treat cardiovascular diseases and vascular dysfunction caused by diabetes (Sun et al., 2017; Su et al., 2022; Huang et al., 2023). In addition, scutellarin also has a protective effect on vascular endothelial cells, preventing hyperglycemia. As Xi et al. (2021) exposed HUVECs to a high-sugar environment to induce *in vitro* vascular endothelial cell damage, and evaluated cell viability by using the MTT assay, Hoechst33342 staining, and flow cytometry to measure the degree of cell apoptosis, using immunofluorescence staining, TEM, and Western blot to determine the changes in mitochondrial autophagic flux, their findings revealed that scutellarin could protect vascular endothelial cells from high-sugar-induced damage by up-regulating mitochondrial autophagy through the PINK1/Parkin signaling pathway, and also provided a theoretical basis and experimental support for scutellarin as a candidate drug for diabetes-induced vascular dysfunction.

## 5 Conclusions and future directions

With the development of modern society, the number of patients with diabetic complications and vascular lesions is increasing. Due to the extremely complex etiology of diabetic vascular lesions, single-treatment methods based on autophagy are often ineffective, while TCM has unique advantages in the treatment of diabetic vascular lesions. There are two main reasons for this phenomenon: on the one hand, since the mechanism of autophagy can involve multiple signaling pathways, it is often difficult for drugs with a single target to consider multiple pathways. Moreover, Chinese herbal medicine can not only play a role through multiple targets, but also consider multiple targets of autophagy in diabetic vascular lesions. On the other hand, Chinese herbal medicine and its active ingredients have been proven to be safe, especially the toxic damage to the liver and kidneys is relatively small. For example, Yigan Mingmu decoction, Danzhi Jiangtang capsules, and Huoxue Jiedu Jiangtang decoction have been used in China for thousands of years. Notably, in view of the TCM preparation in the complex components, cumbersome process and other objective problems, in recent years, many scholars have attempted to separate active ingredients from clinically effective Chinese herbal compound preparations through chemical methods, and apply these active ingredients to cell or animal experiments to elucidate the mechanism of action of Chinese herbal preparations.

At present, there are many shortcomings in the research on autophagy regulation in diabetic vascular lesions in Chinese herbal medicine. The research on autophagy regulation mechanism of diabetic vascular lesions in Chinese herbal medicine is still in the stage of observational research, lacking in-depth exploration of mechanism. This is partly due to the use of natural products in Chinese herbal medicine, which are characterized by confusion. Therefore, more standardization and larger-scale clinical studies are needed to promote the more widespread acceptance of Chinese herbal medicine treatment for patients with diabetic vascular lesions.
